# Spinal cord injury in the setting of traumatic thoracolumbar fracture is not reliably associated with increased risk of associated intra-abdominal injury following blunt trauma: An analysis of a National Trauma Registry database

**DOI:** 10.1016/j.cjtee.2021.03.004

**Published:** 2021-03-26

**Authors:** Veacheslav Zilbermints, Yehuda Hershkovitz, Kobi Peleg, Joseph J. Dubose, Adi Givon, David Aranovich, Mickey Dudkiewicz, Israeli Trauma Group, Boris Kessel

**Affiliations:** a Surgical Division, Hillel Yaffe Medical Center Affiliated to Rappoport Medical School, Technion, Hadera, Israel; b Department of Surgery, Shamir Medical Center Affiliated to Sackler Faculty of Medicine, Tel Aviv University, Tel Aviv, Israel; c National Center for Trauma and Emergency Medicine Research, Gertner Institute for Epidemiology and Health Policy Research, Tel Hashomer, Israel; d University of Maryland School of Medicine, Baltimore, MD, USA; e Hospital Administration, Hillel Yaffe Medical Center, Hadera, Israel

**Keywords:** Spinal fractures, Blunt trauma, Abdominal injuries, Spinal cord injury

## Abstract

**Purpose:**

There is a common opinion that spinal fractures usually reflect the substantial impact of injuries and therefore may be used as a marker of significant associated injuries, specifically for intra-abdominal injury (IAI). The impact of concomitant spinal cord injury (SCI) with the risk of associated IAI has not been well clarified. The aim of this study was to evaluate the incidence and severity of IAIs in patients suffering from spinal fractures with or without SCI.

**Methods:**

A retrospective cohort study using the Israeli National Trauma Registry was conducted. Patients with thoracic, lumbar and thoracolumbar fractures resulting from blunt mechanisms of injury from January 1, 1997 to December 31, 2018 were examined, comparing the incidence, severity and mortality of IAIs in patients with or without SCI. The collected variables included age, gender, mechanism of injury, incidence and severity of the concomitant IAIs and pelvic fractures, abbreviated injury scale, injury severity score, and mortality. Statistical analysis was performed using GraphPad InStat ® Version 3.10, with Chi-square test for independence and two sided Fisher’s exact probability test.

**Results:**

Review of the Israeli National Trauma Database revealed a total of 16,878 patients with spinal fractures. Combined thoracic and lumbar fractures were observed in 1272 patients (7.5%), isolated thoracic fractures in 4967 patients (29.4%) and isolated lumbar fractures in 10,639 patients (63.0%). The incidence of concomitant SCI was found in 4.95% (63/1272), 7.65% (380/4967) and 2.50% (266/10639) of these patients, respectively. The overall mortality was 2.5%, proving higher among isolated thoracic fracture patient than among isolated lumbar fracture counterparts (11.3% *vs.* 4.6%, *p* < 0.001). Isolated thoracic fractures with SCI were significantly more likely to die than non-SCI counterparts (8.2% *vs.* 3.1%, *p* < 0.001). There were no differences in the incidence of IAIs between patients with or without SCI following thoracolumbar fractures overall or in isolated thoracic fractures; although isolated lumbar fractures patients with SCI were more likely to have renal (3.4% *vs.* 1.6%, *p* = 0.02) or bowel injuries (2.3% *vs.* 1.0%, *p* = 0.04) than the non-SCI counterparts.

**Conclusion:**

SCI in the setting of thoracolumbar fracture does not appear to be a marker for associated IAI. However, in a subset of isolated lumbar fractures, SCI patient is associated with increased risks for renal and bowel injury.

## Introduction

Spinal fractures represent 3%–6% of all skeletal injuries accounting more than 160,000 annual spinal injuries in North America.^1^ As a result from high-energy trauma mechanisms such as falls from height and motor vehicle accidents, spinal fractures have a high rate of morbidity and mortality, mostly due to the presence of associated injuries.^2^^,^^3^ A thorough understanding of the epidemiology and concomitant injury patterns better helps guide the evaluation and management of blunt trauma patients from their initial admission to definitive care.

The most common injuries accompanied with thoracolumbar fractures (TLFs) are thoraco-abdominal injuries and pelvic fractures.^4^ Associated spinal cord injury (SCI) with neurologic deficit represents an additional challenge in these patients as, when present, the spinal cord compromise often requires urgent spinal surgery and confounds the ability of physical exam to assist in the diagnosis of associated intra-abdominal injury (IAI).

The purpose of our present study is to determine the impact of SCI in the setting of different types of spinal fractures on the incidence and severity of associated IAIs.

## Methods

This is a retrospective cohort study involving blunt trauma patients with thoracic or lumbar vertebrae fractures or their mixture, from January 1, 1997 to December 31, 2018. Patients with cervical spinal fractures, younger than 18 years of age or without signs of life on arrival to the hospital were excluded. The data were obtained from the records of the Israeli National Trauma Registry maintained by Israel’s National Center for Trauma and Emergency Medicine Research, in the Gertner Institute for Epidemiology and Health Policy Research. The study was approved by Gertner Institute IRB committee (protocol #5138–19, ethical approval number 20187640).

This institute records information concerning trauma patients hospitalized in 19 hospitals, of which 6 are Level I trauma centers and 13 are Level II trauma centers. Data collected in the registry include age, gender, mechanism of injury, incidence and severity of the concomitant IAIs and pelvic fractures, abbreviated injury scale (AIS), injury severity score (ISS), and mortality. Whenever present, parenchymatic abdominal injuries were classified as either minor (The American Association for the Surgery of Trauma (AAST) grade I and II injuries) or significant (moderate and severe; AAST grade III–V). We compared the incidence and severity of abdominal injuries among patients suffering from thoracic and lumbar vertebrae fractures with or without SCI.

Statistical analysis was performed using GraphPad InStat ® Version 3.10 (GraphPad Software Inc., San Diego, CA). Statistical tests performed included Chi-square test for independence and two sided Fisher’s exact probability test. A *p* value of less than 0.05 was considered statistically significant.

## Results

The overall study population included 16,878 blunt trauma patients with different types of spinal fractures. Among these patients, 7.5% (1272) sustained combined thoracic and lumbar fractures, 29.4% (4967) had isolated thoracic fractures and 63.0% (10,639) had isolated lumbar fractures. The incidence of SCI among all trauma victims was 4.2% (709), with SCI documented in the setting of combined thoracic and lumbar fractures being 4.95% (63/1272), 7.65% (380/4967) in isolated thoracic fractures and 2.5% (266/10,639) in isolated lumbar fractures. Table 1 demonstrates the comparison of demographics, injury mechanism severity and mortality outcomes between SCI and non-SCI patients with TLF. The most common mechanism of injury in both groups of TLF patients was fall.

**Table 1:** Demographics, mechanism and severity of 16878 cases of thoracolumbar fractures, *n* (%).

Variables	Thoracolumbar fractures		χ^2^ value	*p* value
	SCI (*n* = 709, 4.2%)	Non-SCI (*n* = 16,169, 95.8%)		
Male gender	503 (70.9)	6159 (61.9)	23.6	0.0001
Age group (years)				0.0001
18-29	229 (32.3)	3529 (21.8)		
30-44	169 (27.6)	3433 (21.2)		
45-59	140 (19.8)	3538 (21.9)	91.05	
60-74	83 (11.7)	3107 (19.2)		
≥75	61 (8.6)	2562 (15.9)		
Mechanism of injury				0.0001
MVA	300 (42.3)	5900 (36.6)		
Fall	325 (45.8)	9213 (57.0)		
Unintentional injury	43 (6.1)	520 (3.2)	58.8	
Intentional injury	37 (5.2)	516 (3.2)		
ISS				0.0001
1-14	258 (36.4)	12447 (77.0)	858.2	
16-75	451 (63.6)	3722 (23.0)		
Mortality	47 (6.6)	366 (2.3)	54.2	0.0001

SCI: spinal cord injury; MVA: motor vehicle accident; ISS: injury severity score.

For all the participants, the most significant percentage of patients had an ISS score of 16 or more. Similar distribution of ISS was observed in patients with isolated thoracic fractures and isolated lumbar fractures as well.

In patients with combined TLF, the mortality rates were found significantly higher in SCI group than in non-SCI group (6.6% *vs.* 2.3%, *p* < 0.0001, Table 1). In examining patients with TLF at isolated thoracic or lumbar locations, a significant mortality increase was observed when associated SCI was noted for isolated thoracic fractures (8.2% *vs.* 3.1%, *p* < 0.001), but not with isolated lumbar fractures (3.0% *vs.* 1.6%, *p* = 0.07).

Among the overall TLF patients with or without SCI, no statistically significant difference was identified either in the total incidence of associated IAIs (15.8% *vs.* 13.75%, *p* = 0.12), or in the incidence of severe (total AIS ≥ 3) injuries (4.8% *vs.* 4.23%, *p* = 0.46).

Dividing TLF into thoracic and lumbar regions, however, did yield some appreciable differences between groups with or without associated SCIs. Whereas there was no difference in the incidence or severity of associated IAIs in thoracic fracture patients (Table 2), lumbar fractures with SCI had significantly higher rates of bowel injury and renal injury, with more severe renal injuries proving more common (Table 3).

**Table 2:** Incidence of associated intra-abdominal injuries in 4967 patients with isolated thoracic fractures, *n* (%).

IAI location	Isolated thoracic vertebral fracture		χ^2^ value	*p* value
	SCI (*n* = 380)	Non-SCI (*n* = 4587)		
Abdomen				
AIS 3-6	22 (5.8)	203 (4.4)	1.50	0.20
Liver	14 (3.7)	117 (2.6)	1.76	0.18
AIS 1-2	8 (2.1)	67 (1.5)	0.98	0.32
AIS 3-6	6 (1.6)	50 (1.1)	0.75	0.38
Spleen	13 (3.4)	150 (3.3)	0.02	0.87
AIS 1-2	6 (1.6)	56 (1.2)	0.36	0.54
AIS 3-6	7 (1.8)	94 (2.0)	0.07	0.78
Kidney	6 (1.6)	68 (1.5)	0.06	0.79
AIS 1-2	3 (0.8)	45 (1.0)	0.13	0.71
AIS 3-6	3 (0.8)	23 (0.5)	0.55	0.45
Diaphragm	2 (0.5)	12 (0.3)	21.5	0.35
Bowel	1 (0.3)	23 (0.5)	12.9	0.52

IAI: intra-abdominal injury; SCI: spinal cord injury; AIS: abbreviated injury scale.

**Table 3:** Incidence of associated intra-abdominal injuries in 10639 patients with isolated lumbar fractures, *n* (%).

IAI location	Isolated lumbar vertebral fracture		χ^2^ value	*p* value
	SCI (*n* = 266)	Non-SCI (*n* = 10373)		
Abdomen				
AIS 3-6	20 (7.5)	555 (5.4)	2.38	0.13
Liver	7 (2.6)	249 (2.4)	0.06	0.80
AIS 1-2	5 (1.9)	162 (1.6)	0.17	0.60
AIS 3-6	2 (0.8)	87 (0.8)	0.02	0.80
Spleen	6 (2.3)	317 (3.1)	0.56	0.45
AIS 1-2	6 (2.3)	131 (1.3)	2.01	0.15
AIS 3-6	0 (0)	191 (1.8)	4.98	0.03
Kidney	9 (3.4)	170 (1.6)	4.77	0.02
AIS 1-2	5 (1.9)	112 (1.1)	1.52	0.20
AIS 3-6	4 (1.5)	59 (0.6)	3.85	0.049
Diaphragm	1 (0.4)	24 (0.2)	0.23	0.60
Bowel	6 (2.3)	102 (1.0)	4.10	0.04

IAI: intra-abdominal injury; SCI: spinal cord injury; AIS: abbreviated injury scale.

## Discussion

Spinal fractures are a common finding in blunt trauma victims, often associated with significant morbidity and potential permanent disability. Approximately half of the spinal fractures identified after these blunt trauma mechanisms are located at thoracolumbar region.^10^ The burden of these injuries has been examined by several previous groups, including one investigation conducted in Germany that identified as many as 8000 severe TLFs occur annually in that country.^11^

It is generally understood that the identification of TLF reflects significant kinetic energy transfer to structures of the human body. Accordingly, it is a common practice that TLF identification warrants a high index of suspicion for the presence of associated injuries to non-spinal structures.^12^ In examinations of this relationship, the association between isolated lumbar fractures and various abdominal injuries has previously been well established.^5^^,^^6^ Bernstein and colleagues^12^ have previously identified that the incidence of IAI in the setting of unstable TLF may be higher than 50%.

SCI also remains a significant potential clinical challenge following blunt injury, although the reported incidence of these injuries varies considerably among existing reports. Hasler and colleagues^13^ in a large cohort European study reported that 1.8% of bony spinal injuries had associated SCI. In another study utilizing the National German Trauma Database and including 57,310 trauma victims, Stephan et al.^8^ demonstrated a five times higher incidence of SCI in a similar study population. The true incidence of SCI is likely to be related to the location and character of bony spinal injury. In case of TVF, the incidence of SCI has been identified as 21.6%, compared to 17.5% among lumbar fracture locations.^14^ In one recent study conducted by several of our present authors using the Israeli National Trauma Database, the incidence of SCI in the setting of TLFs was examined with respect to injury level----finding that SCI occurred in 8.2% of patients with isolated thoracic spinal fractures and 2.5% of those with fractures isolated to the lumbar region.

The identification of SCI in the setting of TLF has the potential to significantly increase the challenges of care for a variety of reasons. First, spinal cord compromise can alter physical exams in detrimental ways. Sensory compromise has the potential to significantly decrease the ability of physical exam to reliably detect regional pain or peritonitis as preliminary indicators of IAI. In addition, patients with SCI and cord impingement due to fracture dislocation are among a subset that may require emergent spinal surgery. The expedient need to decompress the spinal canal may negate the ability to conduct serial exams as a means to detect potential hollow viscus injury that is not clearly manifest on imaging. In this fashion, the presence of SCI in the setting of TLF has the potential to increase the risk for missed or delayed identification of IAIs.

This risk, however, has not been well elucidated. While some investigators have demonstrated that the presence of SCI in the setting of blunt trauma and spinal fracture is indicative of greater kinetic energy transfer^7^^,^^15^ and worse outcome, others have suggested that the detection of a neurologic deficit in these settings does not significantly impact mortality.^8^^,^^9^ As a result, it remains unclear the impact detection of SCI should have on subsequent diagnostic and therapeutic plan development or as a variable to consider with regards to ultimate prognosis. Although the routine utilization of modern medical imaging has almost certainly improved our ability to detect and characterize solid organ injury, the ability to detect occult hollow viscus injuries continues to rely upon the ability of physical exam to augment imaging and laboratory data. An improved understanding of the relationship between SCI in the setting of TLF and hollow viscus injury risk is, therefore, particularly useful.

Our present study suggests that in overall TLF patients, the presence of SCI does not appreciably increase the risk of associated IAI. However, an important caveat manifests when considering isolated thoracic and lumbar fractures specifically. In examining these two distinct subsets, we noted that the presence of SCI significantly increased the likelihood of associated renal and hollow viscus injuries. It is our hope that, armed with this information, practitioners encountering these specific injuries will employ a higher index of suspicion for potential hollow viscus injury accordingly.

To the best of our knowledge, this is the first study to investigate the significance of the presence of SCI, in relation to the incidence and pattern of IAIs. In our opinion, the additional sub-analysis for isolated thoracic and isolated lumbar fractures that we have performed, may have significant impact on the decision making process and warrant further prospective studies.

Our present study has several limitations that must be acknowledged, beginning with the well-known limitations of retrospective design and registry utilization. Specifically, the trauma registry does not include information outlining the specific physical exam findings utilized to identify SCI. The impact of these alterations has significant import on the ability to reliably utilize physical exam. Additionally, the granularity of complications specific to spinal cord injury in registry resources is comparatively limited relative to that afforded by prospective study. For these reasons, additional prospective study on this topic is warranted.

In conclusion, among blunt injured trauma victims with TLFs, the presence of concomitant SCI does not appear to be associated with increased risk for IAI. In a subset of patients with isolated lumbar fractures, however, the presence of SCI is associated with increased risk for both renal and bowel injuries.

## Funding

Nil.

## Ethical statement

The study was approved by Gertner Institute IRB committee (protocol #5138–19, ethical approval number 20187640).

## Declaration of competing interest

The authors declare that they have no known competing financial interests or personal relationships that could have appeared to influence the work reported in this paper.
